# Feedback-related potentials in a gambling task with randomised reward

**DOI:** 10.1016/j.dib.2015.11.060

**Published:** 2015-12-10

**Authors:** Faisal Mushtaq, Pablo Puente Guillen, Richard M. Wilkie, Mark A. Mon-Williams, Alexandre Schaefer

**Affiliations:** a School of Psychology, University of Leeds, Leeds, West Yorkshire, United Kingdom; b School of Computing, University of Leeds, Leeds, West Yorkshire, United Kingdom; c School of Business, Monash University, Sunway Campus, Selangor, Malaysia

**Keywords:** Event-related potentials, Decision-making, Feedback-related, Negativity, Reward processing, Reward prediction error

## Abstract

Event-related potentials (ERPs) time-locked to decision outcomes are reported. Participants engaged in a gambling task (see [1] for details) in which they decided between a risky and a safe option (presented as different coloured shapes) on each trial (416 in total). Each decision was associated with (fully randomised) feedback about the reward outcome (Win/Loss) and its magnitude (varying as a function of decision response; 5–9 points for Risky decisions and 1–4 points for Safe decisions). Here, we show data demonstrating: (a) the influence of Win feedback in the preceding outcome (Outcome_*t*−1_) on activity related to the current outcome (Outcome*_t_*); (b) difference wave analysis for outcome expectancy- separating Expected Outcomes (consecutive Loss trials subtracted from consecutive reward) from Unexpected Outcomes (subtracting Loss_*t*−1_Win*_t_* trials from Win_*t*−1_Loss*_t_* trials); (c) difference waves separating Switch and Stay responses for Outcome Expectancy; (d) the effect of magnitude induced by decisions (Risk*_t_* vs. Safe*_t_*) on Outcome Expectancy; and finally, (e) expectations reflected by response switch direction (Risk to Safe responses vs. Safe to Risk*_t_*) on the FRN at Outcome*_t_*.

**Specifications Table**

**Table:** 

Subject area	*Psychology*
More specific subject area	*Decision-making, Cognitive Neuroscience, Reward Processing*
Type of data	*Event-related potentials time-locked to decision outcome.*
How data was acquired	*Participants completed a two-alternative forced choice decision-making task whilst EEG data were acquired using a 128-channel net connected to a high-input amplifier* (*Electrical Geodesics, Inc., Eugene, OR; see*Fig. 1*for electrode montage) at a rate of* 500 Hz (0.01–200 Hz *bandwidth*) *and an impedance*≤20 kΩ *for frontocentral electrodes. Data were recorded using a Cz reference online and digitally converted to an average mastoids reference offline. After offline filtering* (0.1–30 Hz *bandwidth*)*, data were segmented into* 1000 ms *epochs time-locked to feedback onset* (200 ms *baseline*) *and corrected for artifacts. Values from electrodes were clustered and mean averages from electrodes surrounding the standard FCz location for the FRN and P3a and electrodes surrounding Pz for the P3 were used for statistical analysis.*
Data format	*Analysed ERPs.*
Experimental factors	*Feedback valence was fully randomised.*
Experimental features	*416 trials per participant* (*n*=27).
Data source location	*School of Psychology, University of Leeds, Leeds, United Kingdom.*
Data accessibility	*Within this article.*

**Value of the data**

• Data show feedback-locked potentials on a gambling task when outcomes are fully randomised.
• The FRN, P3a and feedback-related P3 differences for outcome expectancy, switch/stay responses, outcome magnitude and switch direction are reported.
• Results demonstrate that FRN activity does not conform to existing prediction error based accounts.

## 1 Data, experimental design, materials and methods

**Fig. 1:**
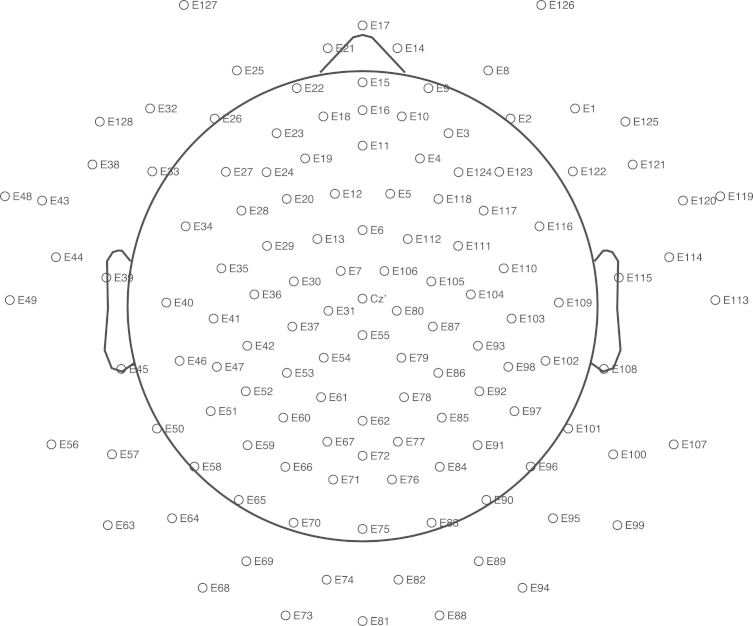
Electrode locations. Schematic representation of the EGI 128 HydroCel^TM^ Geodesic Sensor Net used to acquire EEG data.

EEG data were recorded with a 128-channel net connected to a high-input amplifier (Electrical Geodesics, Inc., Eugene, OR; for electrode montage see Fig. 1) whilst participants engaged in a two-alternative forced choice gambling task (see [1], [2] for further details). On each trial, participants decided between a risky and a safe option, presented as different coloured shapes. In this experiment, gamble outcomes were fully randomised across 416 trials. ERPs time-locked to presentation of feedback were analysed as described in [1]. Values from electrodes were clustered and mean averages from electrodes surrounding the standard FCz location for the FRN (EGI electrode numbers: ‘12’, ‘5’, ‘6’, ‘13’, ‘112’, ‘7’, ‘106’, ‘Cz’, ‘31’, ‘80’ and ‘55’) and P3a and electrodes surrounding Pz for the P3 (EGI electrode numbers: ‘61’, ‘78’, ‘62’, ‘67’, ‘72’, ‘77’, ‘71’ and ‘76’) were used for statistical analysis.

## 2 Reward positivity difference waves

**Fig. 2:**
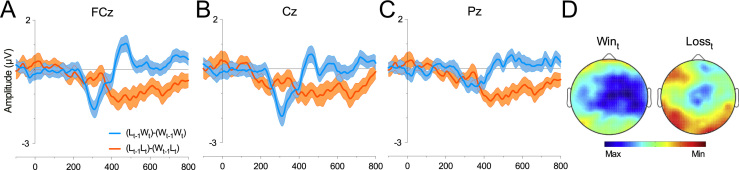
Win*_t_* and Loss*_t_* FRN difference. Difference waveform computed by subtracting Win_*t*−1_Win*_t_* from Loss_*t*−1_Win*_t_* trials (blue) and Loss_*t*−1_−Loss*_t_* from Win_*t*−1_−Loss*_t_* (orange) trials at FCz (A), Cz (B) and Pz (C). Error bars represent SE and abscissa shows time in milliseconds. (D) Topographical maps display mean difference at each electrode site for the FRN time window for Win*_t_* and Loss*_t_* (−1.1 to 0.6 µV).

Difference wave analysis confirmed the FRN was driven by a larger positivity to Win outcomes when preceded by a similarly positive outcome. We found a statistically reliable difference at Outcome_t_ (F [1, 26]=5.81, *p*=.023, *η*^2^_*p*_=.19), with Win*_t_* differences producing greater negativity (*M*=−1.04 µV, SE=.3 µV) than Loss*_t_* differences (*M*=−.04 µV, SE=.3 µV)- complementing a significant peak-to-peak amplitude difference for the same comparison (F [1, 26]=5.23, *p*=.031, *η*^2^_*p*_=.167). Commensurate with the topographical features of the FRN, Fig. 2D shows a frontocentral scalp distribution of this effect. We also performed peak-to-peak analysis to confirm that the Win_*t*−1_Win*_t_* trials were also reliably different from the Loss_*t*−1_Win*_t_* trials (F [1, 26]=9.03, *p*=.006, *η*^2^_*p*_=.258).

## 3 Difference wave analysis: outcome expectancy

**Fig. 3:**
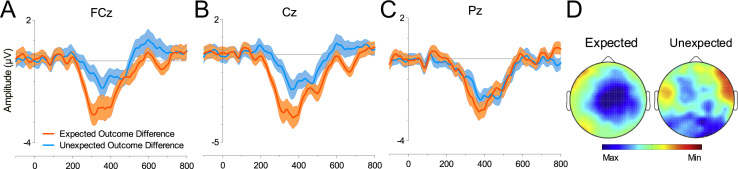
Expected and Unexpected Outcome Differences. (A) FCz, (B) Cz and (C) Pz. Error bars represent SE and abscissa shows time in milliseconds. (D) Scalp maps show the Expected reward difference was maximal at frontocentral in the time-window for the FRN but no such pattern for the difference in Unexpected Outcomes. Maxima and minima specific to each scalp map; Expected: −2.1 to 0.6 µV; Unexpected: −2.7 to 0.69 µV.

The effect of Outcome Expectancy on the FRN was examined by creating two differences waves: (i) Expected Outcome difference wave calculated by subtracting Win_*t*−1_Win*_t_* from Loss_*t*−1_Loss*_t_* trials; and (ii) Unexpected Outcome difference wave calculated by subtracting Loss_*t*−1_Win*_t_* trials from Win_*t*−1_Loss*_t_* trials. Fig. 3 shows a significant difference in FRN activity for Expectancy (F [1, 26]=8.0, *p*=.009, *η*^2^_*p*_=.236), with the Expected Outcome difference producing a larger negativity (*M*=−1.92 µV, SE=.60 µV) than Unexpected Outcome difference (*M*=−0.59 µV, SE=.36 µV).

## 4 Switch vs. Stay: difference wave analysis

### 4.1 Switch vs. Stay: FRN

**Fig. 4:**
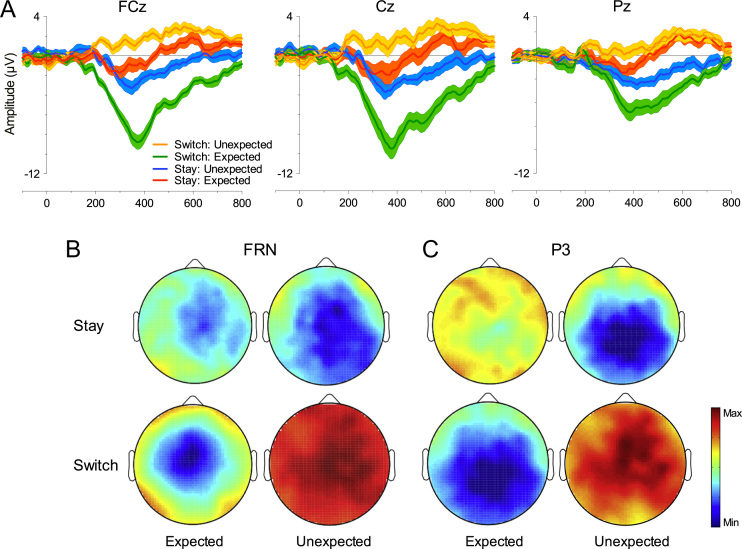
Switch Stay ERPs. (A) ERPs from FCz, Cz and Pz (left to right). Error bars represent SE and abscissa shows time in milliseconds; (B) for the FRN topographical maps show Unexpected Outcomes elicited a frontocentral negativity (left) in Stay trials – consistent with Holroyd and Coles (2002), but this effect was not statistically reliable. The largest difference was observed for Expected Outcomes in Switch trials (maxima and minima: Stay 1.5 to −2 µV; Switch 1.4 to −5.5 µV); (C) for the P3, Unexpected Outcomes for Stay responses and Expected Outcomes for Switch responses produced posteriorly distributed feedback-related P3 differences (maxima and minima: Stay 0.8 to −3 µV; Switch 1.8 to −3.0 µV).

The same approach described in Section 3 was adopted for Switch and Stay trials. There was a main effect of Expectancy (F [1, 26]=25.64, *p*<.0001, *η*^2^_*p*_=.497) and the effect of Strategy approached, but did not reach, significance (F [1, 26]=3.74, *p*=.064, *η*^2^_*p*_=.126). These main effects were qualified by a significant Expectancy×Strategy interaction (F [1, 26]=27.4, *p*<.0001, *η*^2^_*p*_=.513). Visually, consistent with Holroyd and Coles [3], the Unexpected FRN difference (*M*=−1.48 µV, SE=.50 µV) was more negative than Expected (*M*=−0.91 µV, SE=.73 µV) in Stay trials and showed a frontocentral scalp distribution (see Fig. 4B), but this effect was not statistically reliable (F [1, 26]=0.48, *p*=.495, *η*^2^_*p*_=.018). The interaction was driven by Switch trials (F [1, 26]=52.55, *p*<.0001, *η*^2^_*p*_=.669). These trials showed a larger FRN difference for Expected Outcome differences (*M*=−5.12 µV, SE=.66 µV) relative to Unexpected Outcome differences (*M*=.82 µV, SE=.51 µV).

### 4.2 Switch vs. Stay: P3a

For the P3a there was a significant main effect of Expectancy (F [1, 26]=28.91, *p*<.001, *η*^2^_*p*_=.526) and Strategy (F [1, 26]=9.11, *p*=.005, *η*^2^_*p*_=.259) with Switch trials eliciting a greater negativity (*M*=−2.61 µV, SE=.43 µV) than Stay (*M*=−1.11 µV, SE=.43 µV). These effects were qualified by an Expectancy×Strategy interaction (F [1, 26]=46.69, *p*<.0001, *η*^2^_*p*_=.642). The effect of Expectancy approached significance in Stay trials (F [1, 26]=3.57, *p*=.07, *η*^2^_*p*_=.121) with Unexpected Outcomes leading to a greater negativity (*M*=−1.88 µV, SE=.49 µV) than Expected (*M*=−0.35 µV, SE=.73 µV), and was significant in Switch trials (F [1, 26]=58.83, *p*<.0001, *η*^2^_*p*_=.693) with Expected Outcomes leading to a greater negativity (*M*=−6.76 µV, SE=.78 µV) than Unexpected (*M*=1.54 µV, SE=.59 µV).

### 4.3 Switch vs. Stay: feedback-related P3

Consistent with P3a results, there was a significant main effect of Expectancy (F [1, 26]=33.86, *p*<.0001, *η*^2^_*p*_=.566) and Strategy (F [1, 26]=6.87, *p*=.014, *η*^2^_*p*_=.209) with Switch trials eliciting a greater negativity (*M*=−2.62 µV, SE=.44 µV) than Stay (*M*=−1.66 µV, SE=.35 µV). There was a significant Expectancy×Strategy interaction (F [1, 26]=56.11, *p*<.0001, *η*^2^_*p*_=.683). The effect of Expectancy was significant in Stay trials (F [1, 26]=12.95, *p*=.0013, *η*^2^_*p*_=.333) with Unexpected Outcomes differences leading to less positivity (*M*=−2.62 µV, SE=.47 µV) than Expected (*M*=−0.70 µV, SE=.42 µV). There was also a significant effect in Switch trials (F [1, 26]=62.31, *p*<.0001, *η*^2^_*p*_=.706) with Expected Outcome differences leading to less positivity (*M*=−6.01 µV, SE=.74 µV) than Unexpected (*M*=.78 µV, SE=.46 µV).

## 5 P3a analysis

### 5.1 Outcome_*t*−2_×Outcome_*t*−1_×Outcome*_t_*

There was a main effect of Outcome*_t_* (F [1, 26]=4.28, *p*=.049, *η*^2^_*p*_=.141), with Win*_t_* outcomes (*M*=9.72 µV, SE=1.1 µV) relative to Loss*_t_* outcomes (*M*=8.78 µV, SE=1.03 µV). No other effects or interactions approached significance (F׳s<1.99, *p*׳s>.171).

### 5.2 Switch vs. Stay

An Outcome_*t*−1_×Outcome*_t_* ANOVA was conducted for Stay and Switch response trials. For Stay responses, there was no statistically reliable effects or interaction (F׳s<2.6, *p*׳s>.12). For Switch trials, there was a main effects for Outcome*_t_* (F [1, 26]=14.43, *p*<.0001, *η*^2^_*p*_=.357) with more positive going amplitude for Win*_t_* (M=9.01 µV, SE=.96 µV) relative to Loss*_t_* (M=7.46 µV, SE=.88 µV) and for Outcome_*t*−1_ (F [1, 26]=25.42, *p*<.0001, *η*^2^_*p*_=.494): Win_*t*−1_ trials had a greater positive deflection (*M*=9.42 µV, SE=1.1 µV) relative to Loss_*t*−1_ (*M*=7.05 µV, SE=.72 µV). There was also an Outcome_*t*−1_×Outcome*_t_* interaction (F [1, 26]=7.58, *p*=.011, *η*^2^_*p*_=.23), but this effect was qualitatively different to the one observed for the FRN. This interaction was driven by differences at Outcome_*t*−1_ – there was no difference in Outcome*_t_* for Win_*t*−1_ trials (F [1, 26]=.49, *p*=.49, *η*^2^_*p*_=.02), but a substantial effect of Outcome*_t_* when preceded by Loss_*t*−1_ (F [1, 26]=35.60, *p*<.001, *η*^2^_*p*_=.578)- Win trials following Losses (8.37 µV, SE=.85 µV) were larger than sequential losses (5.74 µV, SE=.65 µV).

## 6 Risk-related ERPs

The effect of magnitude on individual ERPs was analysed by conducting an Outcome_*t*−1_×Magnitude×Outcome*_t_* ANOVA for the FRN using a peak-to-peak measure. There was a significant main effect of Magnitude (F [1, 26]=10.19, *p*=.004, *η*^2^_*p*_=.282) but this factor did not interact with Outcomes (F’s<1.36, *p*׳s>.25). Subsequently, difference wave analyses were conducted by separating the ERPs for Outcome*_t_* for the preceding decision (Risk [producing a large outcome] vs. Safe [producing a small outcome]) and valence at Outcome_*t*−1_. Mean amplitude differences were used in a Magnitude (Risk vs. Safe)×Expectancy (Expected Outcome Difference vs. Unexpected Outcome Difference) ANOVA for the FRN, P3a and P3b.

### 6.1 FRN

**Fig. 5:**
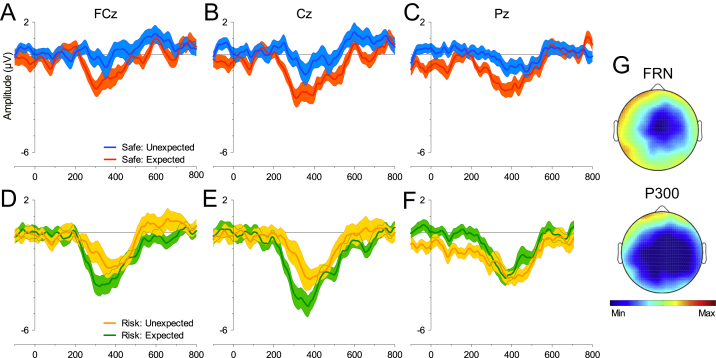
Expectancy and Magnitude. (A–C) ERPs for Expected and Unexpected Reward difference waveforms from FCz, Cz and Pz (left to right) for Safe choices and (D–F) for Safe responses. Error bars represent SE and abscissa shows time in milliseconds; (G) topographical maps show the effect of Magnitude (Safe-Risk) for the FRN and P3 (maxima and minima +1.0 µV to −2.5 µV).

There was a significant main effect of Expectancy (F [1, 26]=9.33, *p*=.005, *η*^2^_*p*_=.264) – with Expected Outcomes giving rise to a greater negativity (*M*=−1.99, SE=.52 µV) than Unexpected (*M*=−.59, SE=.379 µV). There was also a significant main effect of Magnitude (F [1, 26]=5.42, *p*=.028, *η*^2^_*p*_=.172) – with Risk choices differences leading to a larger negativity (*M*=−1.89 µV, SE=.51 µV) than Safe (*M*=−.69 µV, SE=.43 µV). However, Magnitude did not modulate the differences in Outcome Expectancy – as indicated by the Expectancy×Magnitude interaction (F [1, 26]=.015, *p*=.903, *η*^2^_*p*_=.001). To illustrate, difference waveforms for Risk and Safe are plotted separately in Fig. 5.

### 6.2 P3a

There was a significant main effect of Magnitude (F [1, 26]=13.58, *p*=.001, *η*^2^_*p*_=.343) – with Risk choices leading to a larger negativity (*M*=−2.3 µV, SE=.52 µV) than safe (*M*=−.52 µV, SE=.44 µV). However, there was no effect of Expectancy (F [1, 26]=2.78, *p*=.107, *η*^2^_*p*_=.097) and no Expectancy×Magnitude interaction (F [1, 26]=.08, *p*=.783, *η*^2^_*p*_=.003).

### 6.3 Feedback-related P3

There was a significant main effect of Magnitude (F [1, 26]=11.48, *p*=.002, *η*^2^_*p*_=.306) – with Risk choices leading to a larger negativity (*M*=−2.42 µV, SE=.33 µV) than Safe (*M*=−1.35 µV, SE=.33 µV). However, there was no effect of Expectancy (F [1, 26]=1.50, *p*=.232, *η*^2^_*p*_=.054) and the Expectancy×Magnitude interaction did not reach significance (F [1, 26]=1.38, *p*=.251, *η*^2^_*p*_=.05).

## 7 Switch direction×Outcome*_t_* analysis

An Outcome_*t*−1_×Outcome*_t_*×Switch Direction (Risk_*t*−1_Safe*_t_*; Safe_*t*−1_Risk*_t_*) ANOVA was not possible due to a constraint with the number of trials available for this analysis (only 3 subjects had >16 trials across the 8 conditions). In order to examine the impact of expectancy reflected by Switch sequence direction on Outcome*_t_* related activity a Switch Direction×Outcome*_t_* ANOVA was conducted. All 27 subjects had a minimum of 16 artefact-free trials available for this analysis: Risk_*t*−1_Safe*_t_* Loss*_t_* (trials *M*=42, SD=11); Safe_*t*−1_Risk*_t_* Loss*_t_* (trials M=40, SD=9); Risk_*t*−1_Safe*_t_* Win*_t_* (trials *M*=39, SD=8); Safe_*t*−1_Risk*_t_* Win*_t_* (trials *M*=42, SD=9).

**Fig. 6:**
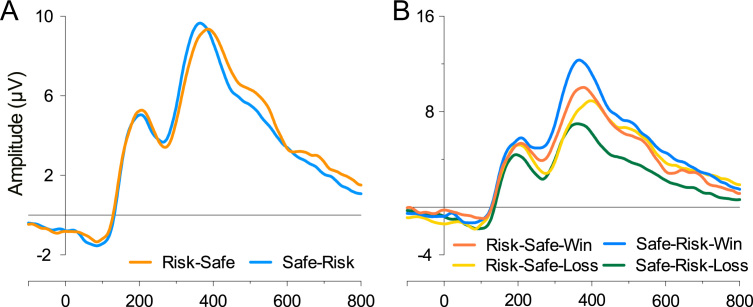
Switch direction and outcome related activity. (A) ERPs at FCz showed marginally significant peak-to-peak FRN effect with greater negativity for Risk_*t*−1_Safe*_t_* responses relative to Safe_*t*−1_Risk*_t_*, however, there was no statistically reliable Switch Direction×Outcome interaction. (B) Abscissa represents time in milliseconds.

There was a main effect of Outcome*_t_* (F [1, 26]=7.05, *p*=.013, *η*^2^_*p*_=.213) and Switch Direction (F [1, 26]=5.38, *p*=.029, *η*^2^_*p*_=.171), with Risk_*t*−1_Safe*_t_* (*M*=−4.45 µV, SE=.72 µV; see Fig. 6A) more negative than Safe_*t*−1_Risk*_t_* (*M*=−3.79 µV, SE=.70 µV). Although visually, the ERPs indicated differences (Fig. 6B), there was no statistically reliable Outcome*_t_*×Switch Direction interaction (F [1, 26]=.01, *p*=.919, *η*^2^_*p*_<.001).
